# Study protocol for a prospective, randomized controlled confirmatory clinical investigation to evaluate the safety and efficacy of a multidisciplinary digital therapeutics in patients with patellofemoral pain syndrome

**DOI:** 10.1186/s13063-025-09030-2

**Published:** 2025-09-01

**Authors:** Tae Hyun Park, Chan Yoon, Jae Hyeon Park, Sanghee Lee, Chi-hyun Choi, Chong Bum Chang, Jin Goo Kim

**Affiliations:** 1 EverEx Inc, 10, Gangnam-daero 94-gil, Gangnam-gu, Seoul, Korea; 2 Department of Rehabilitation Medicine, Hanyang University College of Medicine, Seoul, Korea; 3 Department of Orthopaedic Surgery, Seoul National University Bundang Hospital, Seoul, Korea; 4 Department of Orthopaedic Surgery, Hanyang University Myongji Hospital, Seoul, Korea

**Keywords:** Digital therapeutics (DTx), Patellofemoral pain (PFP), Rehabilitative exercise, Cognitive behavioral therapy (CBT), Software as a medical device (SaMD)

## Abstract

**Background:**

Patellofemoral pain is a prevalent knee condition affecting up to 40% of individuals, especially females aged teens to 50 s. Standard treatments, including exercise therapy, often yield insufficient long-term results, partly due to low compliance and psychological factors like depression and catastrophizing of pain. A digital therapeutics “MORA Cure PFP,” which combines structured progressive exercise and cognitive behavioral therapy via an app, offers a solution to overcome the limitations of conventional treatment for patellofemoral pain patients.

**Methods:**

To evaluate the safety and efficacy of MORA Cure PFP, a two-arm controlled trial will enroll 216 patients diagnosed with patellofemoral pain randomly assigned in a 1:1 ratio to treatment and control groups. The treatment group will use the app, while the control group will perform self-guided exercises using educational materials. This trial aims to determine if the treatment group shows greater reduction in usual pain intensity scores at 8 weeks compared to the control group. Additional assessments include worst pain, knee function, depression, and pain catastrophizing levels.

**Discussion:**

Key design elements of the clinical trial, such as control group selection, inclusion/exclusion criteria, number of patients, and primary endpoint, were designed with consideration for not only medical perspectives but also regulatory aspects of software as a medical device, including device approval and health technology assessment.

**Trial registration:**

ClinicalTrials.gov., NCT06260865, registered 15th February 2024.

**Supplementary Information:**

The online version contains supplementary material available at 10.1186/s13063-025-09030-2.

## Administrative information

Note: the numbers in curly brackets in this protocol refer to SPIRIT checklist item numbers. The order of the items has been modified to group similar items (see http://www.equator-network.org/reporting-guidelines/spirit-2013-statement-defining-standard-protocolitems-for-clinical-trials/).

**Table:** 

Title {1}	**Study protocol for a prospective, randomized controlled confirmatory clinical trial to evaluate the safety and efficacy of a multidisciplinary digital therapeutics in patients with patellofemoral pain**
Trial registration {2a and 2b}	ClinicalTrials.gov., NCT06260865, registered 15th February 2024 (https://clinicaltrials.gov/study/NCT06260865)
Protocol version {3}	The current protocol version is revision 3, dated 20^th^ March 2024.
Funding {4}	This study is funded by EverEx Inc., Seoul, Korea and partially funded by the Ministry of Health & Welfare, Republic of Korea (grant number: RS-2023-KH142023). EverEx is a manufacturer of the digital therapeutic app (medical device) and sponsor of this trial.
Author details {5a}	**Tae Hyun Park**^1^**, MD; Chan Yoon**^1^**, MD; Jae Hyeon Park**^2^**, MD; Sanghee Lee**^1^**, MD; Chi-hyun Choi**^1*^, MD; Chong Bum Chang^3*^, MD; Jin Goo Kim^4^, MD ^1^EverEx Inc., Seoul, Korea ^2^Department of Rehabilitation Medicine, Hanyang University College of Medicine ^3^Department of Orthopaedic Surgery, Seoul National University Bundang Hospital ^4^Department of Orthopaedic Surgery, Hanyang University Myongji Hospital
Name and contact information for the trial sponsor {5b}	EverEx Inc., 29-7 Seoripul-gil, Seocho-gu, Seoul, Korea
Role of sponsor	The study sponsor designed the study, drafted the protocol, conducted review and acceptance from the investigators, obtained approvals from the ministry of food and drug safety (MFDS) and institutional review boards (IRB), and executed funding contracts with the study sites. The sponsor has assigned the contract research organizations (CRO) to perform study monitoring, data management, and statistical analysis. Regarding publication, the sponsor and investigators will jointly publish under mutual agreement. The sponsor plans to apply for device approval from the MFDS based on the clinical trial data.

## Introduction

### Backgroundand rationale {6a}

Patellofemoral pain (PFP) is a common condition with a prevalence ranging from 3 to 40% [1], accounting for 25% of knee pain originating from running [2] and 7 to 10% of all medical consultations [3, 4]. It is more prevalent in females and affects individuals from their teens to their 50 s [1, 3]. The pathophysiology of PFP remains unclear [5], and various non-surgical and surgical treatments have been attempted [6, 7]. Without proper diagnosis and treatment, PFP does not improve spontaneously [8]; over 50% of patients may experience persistent pain for more than 2 years [9], potentially leading to the progression of patellofemoral osteoarthritis [10].

Standard treatment for PFP, according to various guidelines, includes patient education and exercise therapy [11–14]. Strength exercises targeting the quadriceps and hip abductors have proven effective in several studies [15–21]. However, even with evidence-based treatments, 40 to 57% of patients experience insufficient long-term effects [22, 23], highlighting the need for improved therapeutic strategies. Low compliance due to time and location constraints of clinic-based exercise therapy may contribute to this lack of effectiveness [24].

Psychological factors such as anxiety, depression, catastrophizing, and fear of movement are associated with pain and physical function in PFP patients and negatively impact recovery processes like rehabilitation training, as suggested by Maclachlan’s systematic review [25]. To address these factors, Selhorst et al. conducted a randomized controlled trial with adolescent PFP patients, comparing rehabilitation training alone to rehabilitation combined with stepwise cognitive intervention. The group receiving multidisciplinary intervention showed significant improvements in pain and functional levels [26]. Similarly, Bagheri et al. found that young female athletes participating in a mindfulness program alongside exercise exhibited better outcomes in pain severity, knee function, subjective treatment effects, and catastrophizing compared to those who only engaged in exercise [27]. Despite these findings, research on psychological interventions in PFP patients remains limited [26–30], and no studies have combined CBT, which has proven efficacy in chronic pain [31, 32], with exercise therapy in PFP patients.

App-based therapy can overcome time and location constraints of face-to-face treatment, thereby improving patient compliance [33, 34]. EverEx has developed a multidisciplinary treatment app “MORA Cure PFP” for PFP patients that includes self-rehabilitation exercise therapy, which automatically adjusts based on the patient’s status, and CBT for chronic pain. MORA Cure PFP is classified as a software as a medical device (SaMD) that can only be used after diagnosis and prescription by a physician and is prior to regulatory approval. An exploratory clinical trial involving 40 patients, conducted from November 2022 to October 2023 (ClinicalTrials.gov., NCT05614583) demonstrated efficacy and safety comparable to real-world treatment [35].

### Objectives {7}

This confirmatory clinical trial aims to evaluate the safety and efficacy of MORA Cure PFP by comparing it with conventional treatment in PFP patients. For specific objectives, see Table 1.

**Table 1:** Objectives and efficacy endpoints of the trial

Objectives	Endpoints
**Primary** To compare usual pain level at 8 weeks between treatment and control group	Usual pain intensity over the past 1 week at the 8-week (V4)
**Secondary** To compare the following between treatment control group: 1. Worst pain level at 8-week 2. Knee function at 8-week 3. Quality of life at 8-week 4. Health status at 8-week 5. Depression level at 8-week 6. Pain catastrophizing level at 8-week 7. Overall perceived recovery level at 8-week 8. Usual pain at 12-week 9. Worst pain level at 12-week 10. Knee function at 12-week 11. Quality of life at 12-week 12. Health status at 12-week 13. Depression level at 12-week 14. Pain catastrophizing level at 12-week 15. Overall perceived recovery level at 12-week 16. Usual pain level at 4-week 17. Worst pain level at 4-week 18. Knee function at 4-week 19. Change in usual pain level at 8-week compared to baseline 20. Change in worst pain level at 8-week compared to baseline 21. Change in knee function at 8-week compared to baseline 22. Change in quality of life at 8-week compared to baseline 23. Change in health status at 8-week compared to baseline 24. Change in depression level at 8-week compared to baseline 25. Change in pain catastrophizing level at 8-week compared to baseline 26. Change in usual pain level at 12-week compared to baseline 27. Change in worst pain level at 12-week compared to baseline 28. Change in knee function at 12-week compared to baseline 29. Change in quality of life at 12-week compared to baseline 30. Change in health status at 12-week compared to baseline 31. Change in depression level at 12-week compared to baseline 32. Change in pain catastrophizing level at 12-week compared to baseline 33. Change in usual pain level at 4-week compared to baseline 34. Change in worst pain level at 4-week compared to baseline 35. Change in knee function at 4-week compared to baseline	1. Worst pain intensity over the past 1 week at 8-week (V4) 2. Kujala questionnaire total score at 8-week (V4) 3. EQ-5D-5L questionnaire total score at 8-week (V4) 4. EQ-5D-5L questionnaire health status score at 8-week (V4) 5. PHQ-9 questionnaire total score at 8-week (V4) 6. PCS questionnaire total score at 8-week (V4) 7. Overall perceived recovery score at 8-week (V4) 8. Usual pain intensity over the past 1 week at 12-week (V5) 9. Worst pain intensity over the past 1 week at 12-week (V5) 10. Kujala questionnaire total score at 12-week (V5) 11. EQ-5D-5L questionnaire total score at 12-week (V5) 12. EQ-5D-5L questionnaire health status score at 12-week (V5) 13. PHQ-9 questionnaire total score at 12-week (V5) 14. PCS questionnaire total score at 12-week (V5) 15. Overall perceived recovery score at 12-week (V5) 16. Usual pain intensity over the past 1 week at 4-week (V2) 17. Worst pain intensity over the past 1 week at 4-week (V2) 18. Kujala questionnaire total score at 4-week (V2) 19. [Usual pain intensity over the past 1 week at the 8-week (V4)] – [Usual pain intensity over the past 1 week at the baseline (V2)] 20. [Worst pain intensity over the past 1 week at the 8-week (V4)] – [Worst pain intensity over the past 1 week at the baseline (V2)] 21. [Kujala questionnaire total score at the 8-week (V4)] – [Kujala questionnaire total score at the baseline (V2)] 22. [EQ-5D-5L questionnaire total score at the 8-week (V4)] – [EQ-5D-5L questionnaire total score at the baseline (V2)] 23. [EQ-5D-5L questionnaire health status score at the 8-week (V4)] – [EQ-5D-5L questionnaire health status score at the baseline (V2)] 24. [PHQ-9 questionnaire total score at the 8-week (V4)] – [PHQ-9 questionnaire total score at the baseline (V2)] 25. [PCS questionnaire total score at the 8-week (V4)] – [PCS questionnaire total score at the baseline (V2)] 26. [Usual pain intensity over the past 1 week at the 12-week (V5)] – [Usual pain intensity over the past 1 week at the baseline (V2)] 27. [Worst pain intensity over the past 1 week at the 12-week (V5)] – [Worst pain intensity over the past 1 week at the baseline (V2)] 28. [Kujala questionnaire total score at the 12-week (V5)] – [Kujala questionnaire total score at the baseline (V2)] 29. [EQ-5D-5L questionnaire total score at the 12-week (V5)] – [EQ-5D-5L questionnaire total score at the baseline (V2)] 30. [EQ-5D-5L questionnaire health status score at the 12-week (V5)] – [EQ-5D-5L questionnaire health status score at the baseline (V2)] 31. [PHQ-9 questionnaire total score at the 12-week (V5)] – [PHQ-9 questionnaire total score at the baseline (V2)] 32. [PCS questionnaire total score at the 12-week (V5)] – [PCS questionnaire total score at the baseline (V2)] 33. [Usual pain intensity over the past 1 week at the 4-week (V3)] – [Usual pain intensity over the past 1 week at the baseline (V2)] 34. [Worst pain intensity over the past 1 week at the 4-week (V3)] – [Worst pain intensity over the past 1 week at the baseline (V2)] 35. [Kujala questionnaire total score at the 4-week (V3)] – [Kujala questionnaire total score at the baseline (V2)]

### Trial design {8}

**Fig. 1:**
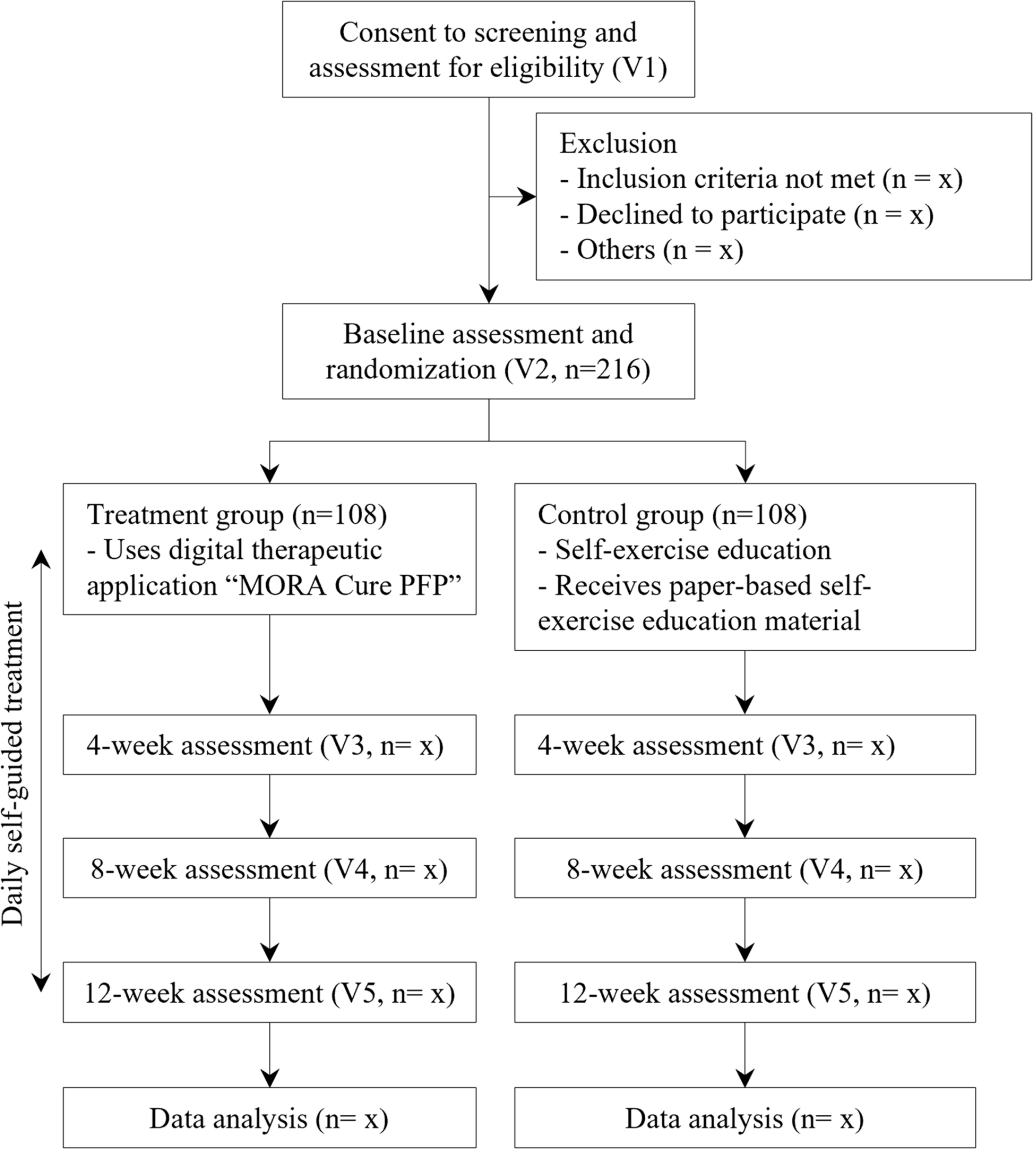
CONSORT flow diagram

This clinical trial is a confirmatory medical device clinical trial and is a two-arm, 1:1 randomized, open-label, multi-centered superiority study. Participants who meet the inclusion/exclusion criteria will be randomly assigned to the treatment group and the control group. Treatment group will be given the digital therapeutic app, and control group will receive exercise education and self-exercise materials. Participant flow diagram according to the Consolidated Standards of Reporting Trials (CONSORT), is presented in Fig. 1.

## Methods: participants, interventions, and outcomes

### Study setting {9}

This clinical trial will be conducted at both academic hospitals and community hospitals (for the list of sites, see https://clinicaltrials.gov/study/NCT06260865) located in South Korea. After being diagnosed and receiving the guidance on the intervention at the hospital, participants will perform the treatment themselves on a daily basis.

### Eligibility criteria {10}

To be included in this study, patients must meet the following criteria: experiencing pain around or behind the patella (kneecap) in unilateral or bilateral knee joints for over 3 months [15, 16, 36]; pain during squatting movements [13, 15–17, 19, 36–39]; pain in two or more of the following activities/tests—prolonged sitting, cycling, running, climbing stairs, kneeling, patellar compression test, tenderness around the patella test [13, 15–17, 19, 36–39]; voluntary decision to participate with signed informed consent.

Exclusion criteria are as follows: knee osteoarthritis exceeding Kellgren-Lawrence (K-L) grade 2 on X-ray [36]; traumatic event causing fractures or dislocations around the knee within the past 3 months [15, 19]; knee surgery within the past 3 months [16, 17, 37, 38]; patellar tendinitis diagnosed by imaging within the past 3 months [37]; current use of narcotic analgesics for pain control [16, 39]; anticipated use of prohibited medications/treatments within 16 weeks of screening; pregnancy; current or recent (within 3 months) participation in another clinical trial; and any other factors deemed unsuitable by the investigator [36].

### Who will take informed consent? {26a}

Before participating in the clinical trial procedures, participants will voluntarily complete and sign an informed consent form. The consent form will be paper-based, and a licensed physician will explain its contents, giving the participant sufficient time to make an informed decision. The consent of the research participants must be obtained in accordance with the ethical principles and standards outlined in the Declaration of Helsinki. All consent forms used will be those approved by the IRB, and a copy of the signed consent form will be provided to the participant. If the consent form is amended, the changes will be explained, and the consent process will be repeated.

### Additional consent provision for collection and use of participant data and biological specimens {26b}

There will be no attempt to retain or request permission from participants to use the data gathered in this study for additional research purposes. No supplementary studies are planned.

This trial does not collect biological specimens.

## Interventions

### Explanation for the choice of comparators {6b}

To evaluate the safety and efficacy of MORA Cure PFP, the treatment group was assigned to use the app. The control group was set as the real-world at best treatment. Standard treatment for PFP, according to various guidelines, includes patient education and exercise therapy [11–14]. Therefore, the control group was defined as those who receive in-person exercise education by healthcare professionals, follow the provided paper-based materials to perform self-exercise, and record their exercise progress.

### Intervention description {11a}

After confirming eligibility based on the inclusion/exclusion criteria, the target knee (left or right) for evaluation will be selected. If only one knee meets the criteria for PFP, that knee will be selected. If both knees qualify, the knee with greater pain will be chosen. All subsequent evaluations will be conducted on the knee selected as the target knee for assessment.

**Fig. 2:**
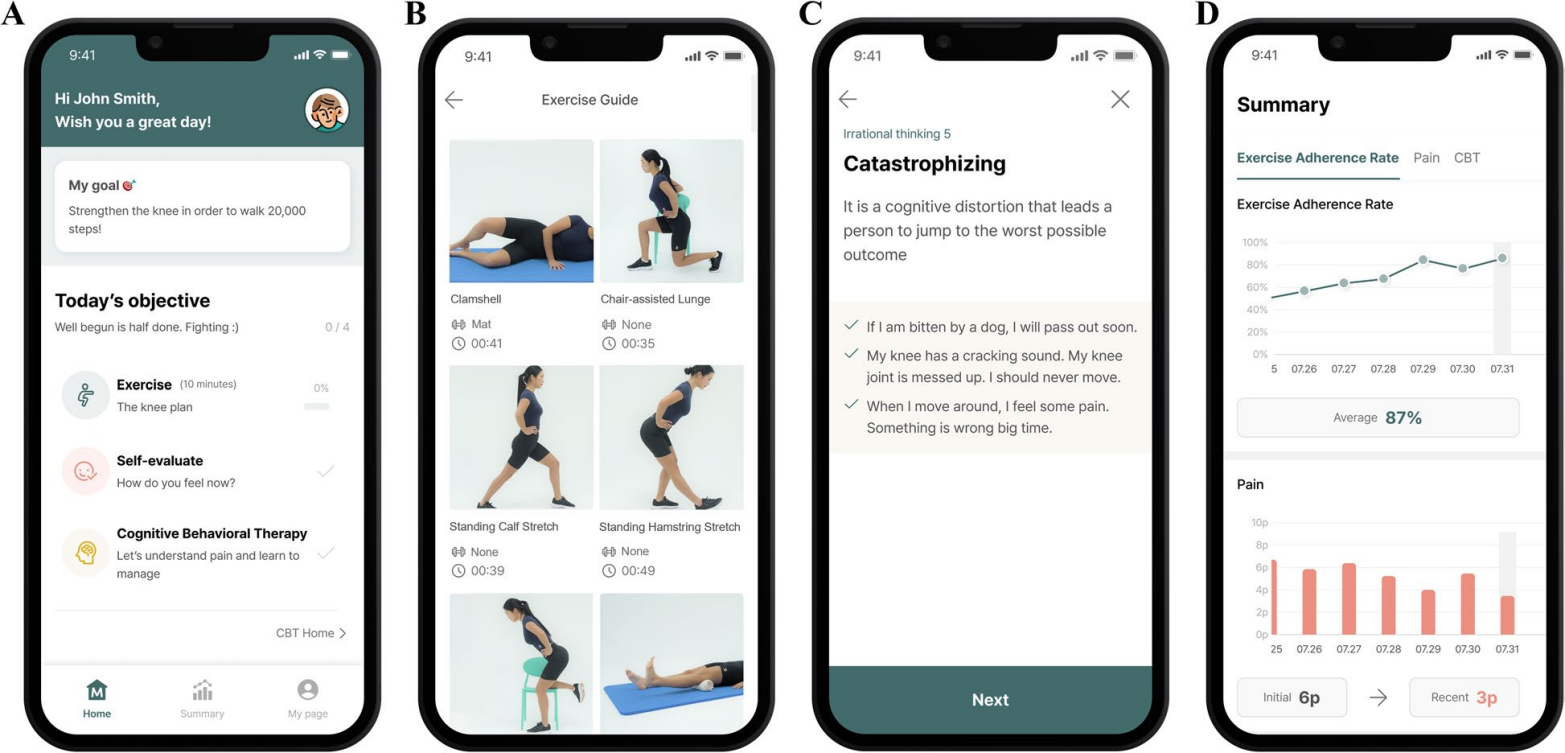
Multidisplicinary digital therapeutic app ‘MORA Cure PFP’ based on self-guided personalized exercise therapy and cognitive behavioral therapy for patellofemoral pain

The investigational medical device “MORA Cure PFP” used in this study will be applied only to participants assigned to the treatment group. MORA Cure PFP consists of a web platform for healthcare professionals and a mobile application for patients. The investigator prescribes treatment through the healthcare web platform, and participants install the application on their mobile phones to begin therapy. At baseline, the investigator will randomize and enroll participants. For those assigned to the treatment group, the investigator will register them on the healthcare web platform and prescribe the device. Upon prescription, an installation code for the patient app will appear on the web platform. Participants will then follow the installation manual to install the app, agree to the terms of service, complete registration, and start the plan, with the Day 1 program beginning on the same day. The device provides an 8-week program of rehabilitative exercise and cognitive behavioral therapy (CBT) for PFP (Fig. 2). After the initial 8-week treatment period, participants can retain access to the device for up to 12 weeks.

For participants in the control group, a healthcare professional with experience in musculoskeletal disease treatment will conduct self-exercise education according to the paper-based self-guided exercise material. The exercise education will last for at least 15 min, and participants will be instructed to record the time spent on self-exercise in the patient diaries distributed at baseline. At each subsequent visit, participants will be asked to bring their ongoing diaries, and whenever feasible, the diaries will be collected to monitor their progress.

### Strategies to improve adherence to interventions {11c}

To enhance adherence, participants are provided with education on the importance of treatment after group allocation. The experimental group receives instructions on using the application, while the control group is taught self-exercise using dedicated materials.

Each participant is required to perform daily self-treatment and log their activities, including exercise completion and duration. For the experimental group, the application automatically tracks and records exercise times, ensuring consistent monitoring. In the control group, participants manually log their exercise in a paper diary. During each visit, the investigator collects the diaries and monitors progress, offering support and feedback to encourage continued adherence.

### Participant timeline {13}

Participants in this clinical trial will complete 4 to 5 outpatient visits: V1 (Screening), V2 (Baseline), V3 (In-treatment), V4 (Post-treatment), and V5 (Follow-up). V2 must occur within 4 weeks of V1 and may be conducted on the same day as V1. V3 is scheduled 4 weeks after V2, with an allowable range of 3 to 5 weeks. V4 is set for 8 weeks after V2, with a window of 8 to 9 weeks, and V5 is scheduled 12 weeks after V2, with an allowable range of 12 to 13 weeks. See Table 2 for the specific schedule of assessments for each visit. For Visit 3, participants may not need to visit the medical institution in person. In this case, certain checks, such as prior medication, concomitant medication, and adverse event assessments, may be omitted. Pain intensity assessment and the Kujala evaluation will be conducted by sending a survey link to the participant, who will complete it while in contact with the clinical trial site representative over the phone.

**Table 2:** Assessment schedule by visit

Schedule	Screening (V1)	Baseline (V2)^1^	In-treatment (V3)^2,3^	Post-treatment (V4)^4^	Follow-up (V5)^5^
	− 4 weeks ~ 0 week	0 week	3 weeks ~ 5 weeks	8 weeks ~ 9 weeks	12 weeks ~ 13 weeks
Informed consent	X				
Screening number assignment	X				
Demographics	X				
Medical history	X				
Height and weight measurement		X			
Physical examination	X	X	X^3^	X	X
Concomitant/previous medication history	X	X	X^3^	X	X
Knee X-ray Imaging and K-L grading	X^6^				
Inclusion/exclusion criteria review	X	X			
Selection of target knee (L/R)		X			
Randomization and assignment of registration number		X			
Pain intensity assessment		X	X	X	X
Kujala questionnaire		X	X	X	X
EQ-5D-5L questionnaire		X		X	X
PHQ-9 questionnaire		X		X	X
PCS questionnaire		X		X	X
Overall perceived recovery score assessment				X	X
Disease education		X			
Investigational device prescription and app installation (treatment group only)		X			
Education on self-exercise (control group only)		X			
Self-exercise patient diary distribution (control group only)		X			
Self-exercise patient diary return (control group only)			X^7^	X^7^	X
Adverse event review		X	X^3^	X	X
Investigational device app deletion (treatment gropu only)					X

1. V2 must be conducted within 4 weeks of V1, and V1 and V2 can take place on the same day

2. V3 is conducted 4 weeks after V2, with a permissible variation of one week before or after (3 to 5 weeks)

3. For V3, a visit to the medical institution may not be required. In this case, previous and concomitant medication reviews, as well as adverse event reviews, may be omitted. Pain intensity and Kujala assessments can be remotely collected by sending a link through a phone call

4. V4 is conducted 8 weeks after V2, with a permissible delay of one week (8 to 9 weeks)

5. V5 is conducted 12 weeks after V2, with a permissible delay of one week (12 to 13 weeks)

6. If an X-ray taken within 3 months is available for K-L grade evaluation, this may be omitted

7. For V3 and V4, the self-exercise log should be reviewed, and completed logs should be collected

### Outcomes {12}

The primary efficacy outcome of this trial is usual pain intensity score (VAS, 100 scale) at 8 weeks (V4). For secondary outcomes, see Table 1. For the method of aggregation, all outcome measures will be analyzed as continuous variables using mean values with appropriate statistical comparisons between groups (For further detail, see Statistical Analysis).

Through interviews, demographic information, medical history, surgical history, and prior/concomitant medications will be collected. Physical examination will include measurements of height, weight, and a patella examination. A knee X-ray will be taken to assign a K-L grade [40], which is used to assess exclusion criteria for osteoarthritis.

The usual and worst pain intensity scores assess the average and peak knee pain experienced over the past week, respectively. Participants mark their pain level on a visual analog scale from 0 to 100, with values recorded and rounded to the nearest whole number [41].

To assess knee function, participants complete the anterior knee pain scale (Kujala score), 13-item self-report questionnaire [42], with a total score calculated from the responses. Quality of life is assessed via the EQ-5D-5L [43] questionnaire, recording the sum of scores across five questions and today’s health status score. Depression severity is evaluated using the Patient Health Questionnaire-9 (PHQ-9) [44], with a total score from nine questions, while pain catastrophizing is assessed using the Pain Catastrophizing Scale (PCS) [45], totaling scores from 13 questions. Participants also rate their perceived recovery level on a 0 to 6 scale [46].

All pain and questionnaire assessments are conducted as electronic patient-reported outcomes (ePRO). Participants use digital tools provided at the site, with data automatically transferred to the electronic case report form (eCRF). For visit 3, if conducted as a televisit, participants may complete the assessments remotely.

### Sample size {14}

The primary efficacy variable of this clinical trial is the intensity of usual pain over the past week at 8 weeks post-treatment initiation, aiming to assess whether the experimental group shows superior improvement compared to the control group. The hypothesis for the superiority test on the primary efficacy variable is as follows: ${H}_{0}: {\mu }_{t}={\mu }_{c} vs. {H}_{1}: {\mu }_{t}\ne {\mu }_{c}$, where ${\mu }_{t}$ represents the usual pain intensity at 8 weeks for the experimental group, and ${\upmu }_{\text{c}}$ represents the same for the control group.

The sample size was calculated based on prior study of MORA Cure PFP (ClinicalTrials.gov., NCT05614583). In the exploratory clinical trial, the usual pain over the past week of full analysis set at 8 weeks post-treatment was 2.31 ± 1.49 for the experimental group and 3.18 ± 2.43 for the control group [35]. The calculated effect size (Cohen’s *d*) is as follows: $d=\frac{{m}_{t}-{m}_{c}}{\sqrt{\left({s}_{t}^{2}+{s}_{c}^{2}\right)/2}}=\frac{2.31-3.18}{\sqrt{\left({1.49}^{2}+{2.43}^{2}\right)/2}}=-0.428$, where ${m}_{t}$ is mean of the experimental group, ${m}_{c}$ is mean of the control group, ${s}_{t}$ is standard deviation of the experimental group, and ${s}_{c}$ is standard deviation of the control group [47]. Values were used up to the second decimal place, and the effect size was calculated without rounding.

Using an effect size of − 0.428, a Type I error of 0.05, and a power of 0.8, the formula for calculating the sample size per group is: $n=\frac{2{\left({z}_{1-\alpha /2}+{z}_{1-\beta }\right)}^{2}}{{\left(d\right)}^{2}}=\frac{2{\left(1.960+0.842\right)}^{2}}{{\left(0.428\right)}^{2}}=86$, where $n$ is required sample size per group (rounded up to the nearest whole number), $\alpha$ is Type I error, $\upbeta$ is Type II error (1 − power), and $d$ is effect size [47].

Considering a typical dropout rate of 10–30% for clinical trials involving digital services [48], a 20% dropout rate was assumed for this study. Adjusting for this dropout rate, the required sample size per group is calculated as: ${n}^{\text{'}}=86\times \frac{1}{1-r}=107.5$, where *r* is the dropout rate (0.2). Thus, the target sample size is set at 216 participants in total, with 108 per group (Fig. 1).

### Recruitment {15}

Participant recruitment will be handled by the institution. The investigator may invite patients under their care to participate, or participants may be recruited through in-hospital posters or an internet page. In these cases, participants must visit the institution to complete the informed consent form and undergo an eligibility check. All recruitment methods will be implemented only after obtaining IRB approval.

### Criteria for discontinuing or modifying allocated interventions {11b}

Participants will be withdrawn from the trial if they receive unapproved medication, procedures, or treatments that could impact trial outcomes; fail to meet inclusion/exclusion criteria post-enrollment; are lost to follow-up; experience adverse events requiring withdrawal as determined by the investigator; request to discontinue (withdrawal of consent); or if the investigator deems their continued participation unsuitable for any other reason.

If a participant misses a visit, the investigator must make every effort to contact them, encourage attendance, confirm their well-being, and document the reason for absence. Withdrawn participants will be included in efficacy and safety evaluations unless a valid reason for exclusion exists. Modifications to the intervention are not allowed in this trial.

### Relevant concomitant care permitted or prohibited during the trial {11d}

During the clinical trial, participants may use acetaminophen 650 mg as a rescue medication if they experience intolerable pain. The investigator may prescribe this at visits. Participants already on pain medication before the trial are encouraged to switch to the rescue medication but may continue their existing one at the investigator’s discretion. From baseline (visit 2) to follow-up (visit 5), participants should ideally avoid pain medications within 24 h before each visit.

The following are prohibited from visit 1 to visit 5: intra-articular or knee injections (e.g., corticosteroids), arthroscopic surgery, corrective osteotomy, knee replacement, or any unapproved procedures or surgeries, opioid analgesics for pain, topical knee ointments or patches, unapproved joint pain medications or treatments, and high-impact physical activities.

### Provision for post-trial care {30d}

After completing or withdrawing from the clinical trial, participants may resume regular medical care as before. If an adverse device effect arises, they may be compensated for medical expenses per the compensation policy until the issue is resolved. The sponsor will secure IRB approval for the insurance policy covering such compensation. Additionally, if there is any suspicion of an adverse event after a participant’s trial completion or withdrawal, the investigator must assess and provide further medical care as needed.

Participants will not be able to continue accessing the app after the trial period ends. The investigational medical device will be deleted from participants’ mobile phones at V5 (end of trial period), as access to unapproved medical devices by study participants must be limited to the minimum necessary for the trial.

## Assignment of interventions: allocation

### Sequence generation {16a}

Only participants who meet the inclusion/exclusion criteria will be randomly assigned to either the experimental or control group in a 1:1 ratio. The method used for this assignment is computer-generated block randomization. To ensure independence, a third-party institution will be entrusted with generating the randomization list. Stratification will not be performed.

### Concealment mechanism {16b}

To conceal the randomization list, neither the sponsor nor the delegated CRO will have access to the list. The sequence will be generated within the third-party institution, and it will be directly built into the eCRF system. The results of this process will be encrypted and will not be identifiable.

### Implementation {16c}

Random assignment will be conducted through an interactive web response system (IWRS) incorporated into eCRF. The pre-generated randomization list will be embedded into the program.

## Assignment of interventions: blinding

### Who will be blinded {17a}

This clinical trial is open-label; therefore, blinding is not applicable.

Due to the nature of digital healthcare apps, complete blinding of participants and investigators is not feasible. Statistical analysis by the biostatistician will commence only after trial completion and proper verification of all data followed by database lock. (For further detail, See “Data management {19}”). The additional benefit of blinding the biostatistician is limited.

### Procedure for unblinding if needed {17b}

This clinical trial is open-label; therefore, blinding is not applicable.

## Data collection and management

### Plans for assessment and collection of outcomes {18a}

Source documents refer to all original records of clinical findings, observations, or other activities essential for the evaluation and replication of the clinical trial. Examples include, but are not limited to, medical records, clinical findings, knee X-rays for K-L grade evaluation, participant diaries for recording treatment compliance, and patient-reported outcomes collected via electronic devices (pain intensity, Kujala scale, EQ-5D-5L, PHQ-9, PCS, and overall perceived level of recovery). For the treatment group, compliance data from the application can also serve as source data.

Data collection and recording are performed by the investigator under the supervision of the principal investigator, electronic CRF as the primary method of data collection. The investigator must ensure the accuracy, completeness, legibility, and timeliness of the data included in the case reports and other records. Data recorded in the CRF must be consistent with the source documents; any discrepancies or omissions must be accompanied by an explanation. Changes to electronic CRFs must be traceable through an audit trail system.

### Plans to promote participant retention and complete follow-up {18b}

Participants will receive 50,000 won (equivalent to 26 US dollars) per visit as transportation reimbursement. If Visit 1 and Visit 2 are conducted simultaneously, participants will receive double the amount, and the same amount will be provided for Visit 3, even if it is an electronic visit. At each visit, the investigator will check and monitor the treatment progress (automated data from the app for the treatment group, patient diaries for the control group). The application includes a notification feature that allows reminders to be sent at the patient’s preferred time, and personalized messages are sent based on exercise compliance to help motivate the patient.

To meet regulatory requirements and ensure study integrity, data from participants who leave or are withdrawn from the trial will be collected, recorded, and retained for the period during which they participated in the study.

### Data management {19}

All clinical trial sites and investigators must ensure participant confidentiality and maintain accurate, legible, and timely records in compliance with regulations. The sponsor and regulatory authorities must be granted access to review records for monitoring, audits, and inspections. When using eCRF, authorized investigators enter data and automated systems detect discrepancies, issuing queries for corrections or confirmations. If IWRS or ePROs are used, data integration with the eCRF may occur.

The sponsor reviews the eCRFs for completeness and may issue queries for discrepancies or missing data, which the investigator must address. After the trial, protocol violations are determined, and once data integrity is confirmed, a database lock (DB lock) is performed, and a copy of the participant database is sent to the investigator. Statistical analyses take place post-DB lock.

The investigator and trial site must retain essential documents for a minimum of 3 years as required by country law, with the sponsor specifying the retention period. If further retention is unnecessary, the sponsor must inform the investigator and the clinical trial site in writing.

### Confidentiality {27}

The investigator must ensure the anonymity of study participants, avoiding any identification of participants’ names in documents submitted to the sponsor. Signed consent forms, source documents, and participant logs must be kept confidential and stored securely. All personal information collected through the investigational device follows personal information processing policy, and participants shall consent before installing the app.

Data collection systems in this trial comply with US code of federal regulation chapter 21, part 11 and local regulations. All data transmissions are encrypted, and access is controlled through individual user IDs and passwords, with only authorized and trained personnel permitted access.

### Plans for collection, laboratory evaluation, and storage of biological specimens for genetic or molecular analysis in this trial/future use {33}

Not applicable as no biological samples are to be collected.

## Statistical methods

### Definition of analysis sets

In this clinical trial, data will be analyzed using three sets: the full analysis set (FAS), the per protocol set (PPS), and the safety set. The FAS, aligned with the intention-to-treat (ITT) principle, will be the primary analysis set for efficacy evaluations, with additional analyses using the PPS. If discrepancies arise between the two, the outcomes will be presented and investigated. Safety evaluations will use the safety set.

The FAS includes participants who meet all of the following: have post-randomization primary efficacy data, records of applying the investigational device (test group) or performing self-exercise (control group), and were not improperly enrolled due to inclusion/exclusion violations. The PPS is a subset of the FAS, including those who completed the trial without major protocol violations, did not withdraw prematurely, and had an overall compliance rate of 50% or higher. The safety set includes all randomized participants with records of using the investigational device or performing self-exercise.

### Methods in analysis to handle protocol non-adherence and any statistical methods to handle missing data {20c}

For the analysis of the FAS, missing values occurring after treatment that are used for primary and secondary efficacy evaluation variables will be imputed using the last observation carried forward (LOCF) method. However, when imputing missing values, results prior to the administration of the investigational medical device will not be substituted for post-administration results. Other data will be analyzed as collected (available data) without imputation.

For the analysis of the PPS, missing values will not be imputed and will be analyzed as collected.

### Statistical methods for demographic and other baseline characteristics

The full analysis set (FAS) will be analyzed for demographic information, medication history, treatment history, and past/current medical history of participants. Categorical data (e.g., gender) will be presented as frequency and percentage within the safety population, while continuous data (e.g., age) will be summarized using descriptive statistics, including mean, standard deviation, median, minimum, and maximum values. Results will be reported separately for the experimental and control groups. Group comparisons will use a two-sample *t*-test for continuous variables (or Wilcoxon’s rank-sum test if normality is not met) and Pearson’s chi-square test for categorical variables (or Fisher’s exact test if more than 20% of cells have expected counts below 5).

Past and current medical history will be standardized using the latest the Medical Dictionary for Regulatory Activities (MedDRA) version, classified by system organ class (SOC) and preferred term (PT), with frequencies and percentages reported per group. Medication and treatment history will be standardized by the latest anatomical therapeutic chemical (ATC) classification, reported by level 1 (anatomical main group) and level 2 (therapeutic subgroup), and analyzed by Pearson’s chi-square test (or Fisher’s exact test where appropriate).

### Statistical methods for primary and secondary outcomes {20a}

For the definition of efficacy evaluation variables, see Table 1. Descriptive statistics (mean, standard deviation, median, minimum, maximum) will be presented for both experimental and control groups. Group differences will be analyzed using ANCOVA with baseline values as covariates, providing adjusted means (least square means), *p*-values, and 95% confidence intervals. Statistical tests will be two-sided with a significance level of 0.05, and results will include clinical interpretation.

Safety analysis will be conducted on the safety set. Adverse events (AEs) will be standardized by SOC and PT using the latest MedDRA version. Treatment-emergent adverse events (TEAEs) will be recorded as a single event per participant if they share the same SOC and PT, with severity classified by the highest severity reported, and causal relationship classified by the stronger link to the investigational device. Analysis will focus on TEAEs, including counts and percentages for TEAEs, knee-related TEAEs, adverse device effects (ADEs), and serious adverse events (SAEs). Percentage differences will be tested using Pearson’s chi-square test or Fisher’s exact test, if appropriate.

### Interim analyses {21b}

There are no interim analyses planned.

#### Methods or additional analyses (e.g., subgroup analyses) {20b}

To determine whether the results of the primary efficacy evaluation differ according to subgroups of study participants, a subgroup analysis will be performed. The subgroup analysis will compare participants with a usual pain intensity score over the past week at visit 2 of 50 points or higher to those with a score of 49 points or lower.

### Plans to give access to the full protocol, participant-level data, and statistical code {31c}

There are no plans to give access to the full protocol, participant-level data, and statistical code.

## Oversight and monitoring

### Composition of the coordinating center and trial steering committee {5d}

The investigators will hold investigator meetings as needed, led by two experienced orthopedic surgeons who have been assigned the role of coordinating investigators. Information regarding the protocol and the device must be provided to the investigators prior to the start of the trial, and formal acceptance must be obtained. Before the trial begins, the sponsor will visit each site for a site initiation visit (SIV), where the research team will be briefed on the trial, and the final operational details will be confirmed. If necessary, a site feasibility meeting may be conducted prior to the SIV meeting.

The sponsor and CRO will hold monthly meetings to review the progress of the study and discuss any issues that arose during routine monitoring visits. If necessary, additional QC visits, individual investigator meetings, or investigator group meetings may be scheduled to address specific concerns or ensure the smooth continuation of the trial.

### Composition of the data monitoring committee, its role and reporting structure {21a}

This study does not include a data monitoring committee (DMC). The decision to exclude a DMC is based on the fact that the study involves minimal risk to participants, and the investigational medical device has already demonstrated a favorable safety profile in prior studies. Additionally, the study design allows for close monitoring by the investigators and sponsor through regular site visits and data reviews, ensuring that participant safety and data integrity are adequately maintained without the need for an independent monitoring committee.

### Adverse event reporting and harms {22}

All adverse events that occur or worsen after obtaining the subject’s consent will be collected, except for those who fail the screening. Pre-existing signs or symptoms at the time of consent are recorded in the medical history section of the CRF. AEs should be monitored until symptoms resolve and the condition stabilizes, and progress reports may be required by the sponsor. The investigator should provide a clear diagnosis, or otherwise record symptoms/signs. Appropriate examinations and treatments must be administered for all AEs. If an AE makes it difficult to continue the trial, the subject may drop out or the trial may be discontinued, with the best treatment provided for safety.

The CRF must record whether the AE is treatment-emergent (TEAE), the onset and end dates, severity, causal relationship with the investigational device, actions taken, and outcome. Serious adverse events (SAEs), serious adverse device effects, and unexpected adverse device effects must be reported to the IRB and regulatory authorities within the required timeframes. Specific AEs, such as worsening knee pain, cramps, dizziness, changes in mood or thought patterns, and any other significant AEs, must be documented and shared with the sponsor within 24 h.

All adverse events that occur will be standardized using System Organ Class (SOC) and Preferred Term (PT) with the latest version of MedDRA. Treatment emergent adverse events (TEAEs) that occur after application of the investigational medical device will be counted as one event when the same SOC and PT adverse event occurs multiple times in one subject, and when the severity differs for the same adverse event, it will be handled with the maximum severity. When the causal relationship differs for the same TEAE in one subject, it will be handled in the direction of higher relatedness to the investigational medical device. Summary and analysis of adverse events will be performed for TEAEs. The number of subjects experiencing events, incidence rates, and number of events will be presented for TEAEs, knee-related TEAEs, ADEs, and SAEs.

### Frequency and plans for auditing trial conduct {23}

Before the clinical trial begins, the sponsor and investigator review the protocol and CRFs during a site initiation visit or investigator meeting. Monitoring is conducted by the sponsor or their representative, who regularly visits the site to verify the completeness of source documents, the accuracy of case records, adherence to Good Clinical Practice (GCP) and the protocol, participant enrollment, and the management of investigational devices and supplies. Monitors discuss findings with key investigators and ensure that source documents and CRFs are consistent, especially regarding consent, inclusion/exclusion criteria, and SAEs.

The investigator must retain all source documents, including signed consent forms, and ensure data traceability. Monitoring follows the sponsor’s procedures, with results reported to the sponsor. Audits are performed by the sponsor, while the MFDS conducts inspections. If notified of an MFDS inspection, the investigator must inform the sponsor promptly.

### Plans for communicating important protocol amendments to relevant parties (e.g., Trial participants, ethical committees) {25}

When changes to the approved clinical trial protocol are needed, approval must be obtained from the IRB and MFDS. The sponsor and investigator must document their agreement to follow the protocol, including any amendments, with signatures and dates. The investigator cannot deviate from the protocol without prior approval from the sponsor, IRB, and MFDS, except in cases where immediate action is required to protect participants. In such emergencies, the investigator must report the changes, along with reasons, to the sponsor, IRB, and MFDS as soon as possible to obtain retroactive approval.

### Dissemination plans {31a}

Regardless of the study results, whether positive or not, the sponsor and investigators have the right to publish or present any data collected or generated from the clinical trial. If the investigators wish to present or publish the study results externally, it must first provide the sponsor with an opportunity to review the manuscript or any other disclosure materials in advance to prevent the inadvertent release of confidential information or inventions, and obtain approval before publication or disclosure.

## Discussion

This clinical trial is a double-arm randomized controlled trial aimed at evaluating the safety and efficacy of the digital therapeutic app for PFP (MORA Cure PFP). The app used in the trial includes guideline-based exercise therapy and CBT, designed to facilitate high levels of patient interaction and features suitable for self-guided use, which are expected to improve treatment compliance. The intervention in this study will provide a new method for the rehabilitation of patients with PFP.

In recent years, digital therapeutics have gained significant attention. Digital therapeutic devices are primarily applied to chronic conditions such as diabetes or psychiatric disorders, and useful in correcting lifestyle and behavioral habits. Recently, digital therapeutics targeting musculoskeletal diseases have been under development, and results from a clinical trial for one targeting fibromyalgia have been published [49].

From a regulatory perspective, digital therapeutics are classified as software as a medical device (SaMD) [50]. SaMD refers to software that qualifies as a medical device on its own, as opposed to hardware-based medical devices. Digital therapeutics are specifically designated as treatment-focused SaMDs, which require objective demonstration of therapeutic effectiveness to gain regulatory approval. Prospective clinical trials serve not only as an objective process for regulatory approval by observing therapeutic effects but also as valuable data for subsequent health technology assessments and insurance reimbursement listings. Therefore, when designing clinical trials for digital therapeutics, considerations should include not only medical aspects but also regulatory approval and insurance listing perspectives for medical devices.

In designing clinical trials for digital therapeutics, specific considerations are essential. For instance, some trials use a “sham device,” a simplified app lacking therapeutic features, as the control group [51]. However, this trial aims to demonstrate the app’s superiority over the at-best treatment (treatment-as-usual). Therefore, the control group will receive in-person exercise education with materials and resources for self-guided exercise without app use. The inclusion/exclusion criteria should accurately represent the indication. In this trial, PFP guidelines [11–14] are used to select participants with chronic patella-related pain lasting over 3 months and confirm objective pain elicited through movement or physical exams. Given that PFP is often diagnosed by exclusion [11–14], patients with conditions such as osteoarthritis, fractures, dislocations, surgical history, or patellar tendinitis are excluded. Additionally, patients requiring high-potency drugs like narcotic analgesics are beyond the app’s intended use and are excluded. The required participant number is designed based on the app’s feasibility trial [35], offering a more objective basis than calculations based on non-app studies. As the name implies, PFP is characterized primarily by pain, so the primary endpoint is the usual pain level at 8 weeks, with expectations that the app group will report lower pain levels than the control group.

A representative digital therapeutic for knee-related conditions is Mawendo (manufactured by Mawendo GmbH, Germany). In Germany, digital therapeutics are covered under the category of Digitale Gesundheitsanwendungen (DiGA) by public health insurance (Gesetzliche Krankenversicherung, GKV), and Mawendo accounts for approximately one quarter of the total reimbursement within the DiGA category [52]. A randomized controlled trial involving 259 participants showed statistically significant therapeutic superiority of Mawendo over standard physiotherapy [53]. Its primary outcomes are functional improvement, as measured by knee injury and osteoarthritis outcome score-activities of daily living subscale (KOOS-ADL), and pain reduction, assessed using VAS. While Mawendo’s typical treatment period spans 12 weeks, this study will include an 8-week treatment phase followed by a 4-week extra curriculum. An additional difference of this study is its inclusion of psychological measures, evaluating depression and pain catastrophizing levels.

## Limitations

There are several limitations in this trial. First, for the treatment group, the placebo effect of the “app” itself, rather than the therapeutic program, may be considered. However, given that app-based therapy inherently increases interaction and compliance, this should be seen as a contributing factor to the treatment rather than a placebo. Additionally, when evaluating the device—especially in value-based health technology assessments—comparison with the current at-best therapy is more appropriate. Second, the assessment tools used in this trial, including pain measured with the VAS scale and various questionnaires, lack objectivity. However, given that the evaluation is not based on objective lab values but on assessments of the symptoms, some subjectivity in evaluation is inevitable. Furthermore, since there are no direct comparison targets such as “exercise-only” or “CBT-only” apps, it is challenging to clarify which specific component plays a primary role. Finally, blinding was not feasible in the design of the study’s experimental and control groups, which may be vulnerable to bias.

## Conclusion

The digital therapeutic app for PFP offers a structured progressive exercise program and CBT designed for chronic pain, with the goal of enhancing compliance and treatment effectiveness. This trial will evaluate the safety and efficacy of the digital therapeutic app compared to an active control (treatment as usual).

## Trial status

The current protocol version is revision 3, dated 20th March 2024. Participant recruitment began on 26th April 2024 and is expected to be finished by the end of December 2024.

## Supplementary Information

Supplementary Material 1.

## Data Availability

All intangible and tangible products resulting from the conduct of this study, including all reports, data, attached documents, work outcomes, and intellectual property, shall be owned by the sponsor. This provision is ensured through a documented agreement among the sponsor, investigator, and site, established before the commencement of the clinical trial.

## Abbreviations

ADE: Adverse device effect
AE: Adverse event
ATC: Anatomical therapeutic chemical
CBT: Cognitive behavioral therapy
CRF: Case report form
CRO: Contract Research Organization
DB: Database
eCRF: Electronic case report form
ePRO: Electronic patient-reported outcome
FAS: Full analysis set
GCP: Good Clinical Practice
ITT: Intention-to-treat
IRB: Institutional Review Board
LOCF: Last Observation Carried Forward
MedDRA: The Medical Dictionary for Regulatory Activities
MFDS: Ministry of Food and Drug Safety
PCS: Pain Catastrophizing Scale
PFP: Patellofemoral pain
PHQ-9: Patient Health Questionnaire-9
PP: Per Protocol
PPS: Per Protocol Set
PT: Preferred term
SAE: Serious adverse event
SaMD: Software as a Medical Device
SOC: System organ class
TEAE: Treatment-emergent adverse event
VAS: Visual analog scale
