# Supplementary material for: Study protocol for a prospective, randomized controlled confirmatory clinical investigation to evaluate the safety and efficacy of a multidisciplinary digital therapeutics in patients with patellofemoral pain syndrome
Source: Trials. 2025 Sep 1;26:328. doi: 10.1186/s13063-025-09030-2 (PMC12403444; doi:10.1186/s13063-025-09030-2)
Supplement: Supplementary file 1 — Supplementary Material 1. [file 13063_2025_9030_MOESM1_ESM.pdf]

연구대상자 설명문

1 임상시험 정보

임상시험 제목	슬개대퇴통증증후군의 치료를 위해서 사용되는 다학제적 디지털치료기기의 안전성과 유효성을 평가하기 위한 전향적, 무작위배정 확증 임상시험
계획서 번호	E-ETH-01K-CI02
의뢰자	에버엑스 주식회사

시험책임자	공란
시험책임자의 소속	공란
주소	공란
연락처	공란

2 서론

본 임상시험 참여 결정은 귀하의 자발적 동의를 통해 이루어지며, 언제든지 연구 중단을 결정할 수 있고 임상시험과 관련된 질문이 있는 경우 자유롭게 문의할 수 있습니다. 귀하는 임상시험 참여 여부를 결정하기 위해 충분한 시간을 가질 수 있습니다. 귀하가 임상시험에 참여하지 않더라도 어떤 불이익도 입지 않을 것이며, 향후 진료/치료 과정에 어떠한 영향도 미치지 않을 것입니다.

3 임상시험에 대한 설명

3.1 임상시험의 배경 및 개발 경위

슬개대퇴통증증후군은 30~40%의 높은 유병율이 보고되는 흔한 질환입니다. 전체 병원을 찾는 환자 중 7~10%가 이에 해당한다는 보고도 있습니다.

슬개대퇴통증증후군의 기존 치료는 약물 치료, 물리 치료, 통증의 관리 등에 초점을 맞추어 왔습니다. 하지만 최신의 치료 가이드라인에 의하면 약물 치료와 더불어 심리적 치료, 운동 치료 등을 함께 시행하는 다학제적(multidisciplinary) 접근이 필요합니다. 최신의 논문 근거에 의하면 인지행동치료(cognitive behavioral therapy, CBT)와 같은 심리적 치료와 운동 치료를 함께 제공할 때 통증 완화와 신체 기능 강화에 더 효과적이라는 것이 보고된 바 있습니다.

기존의 운동치료 또는 인지행동치료는 환자가 병원에 방문하여 의사 또는 물리치료사 등을 전문가를 대면하여 시행하는 것이 대부분이었습니다. 하지만 병원을 방문하여 여러 가지 치료를 받기에 애로 사항이 많고, 환자의 시간적/금전적 부담도 과중하였습니다. 따라서 치료 효과를 높이기 위해서는 운동치료와

인지행동치료의 접근성을 높일 필요가 있으며, 이는 휴대폰과 어플리케이션, 인공지능 기술 등을 이용하여 이루어질 수 있습니다. 본 임상시험에서 사용되는 임상시험용 의료기기 MORA Cure는 디지털치료기기(digital therapeutics, DTx)로 재활운동치료와 함께 인지행동치료가 적용되었고, 비대면 방식으로 치료를 수행하게 됩니다. 이에 따라 환자의 시간적/금전적 부담은 줄어들고 치료의 효과는 높아질 것으로 예상됩니다.

3.2 임상시험의 목적

본 임상시험의 목적은 슬개대퇴통증증후군 환자에서 재활운동 치료와 인지행동치료가 적용된 디지털치료기기 MORA Cure의 안전성 및 유효성을 평가하는 것입니다.

본 임상시험은 연구 목적으로 수행되며 검증되지 않은 의료기기를 사용합니다. 본 임상시험에 사용되는 의료기기는 아직 허가를 받지 않았으며, 본 임상시험은 식품의약품안전처의 허가를 받기 위한 임상시험입니다.

3.3 임상시험용 의료기기에 관한 정보

본 임상시험에 사용되는 의료기기 MORA Cure는 연구대상자가 보유한 스마트폰에 설치하여 사용하는 모바일 어플리케이션입니다. 총 8주까지 재활운동 프로그램 및 인지행동치료 프로그램이 모바일 어플리케이션을 통해 제공되며, 치료 후에도 12주차까지 어플리케이션을 사용할 수 있습니다.

귀하의 휴대폰에 의료기기를 설치하고 정상적으로 작동하기 위해서는 다음의 최소 사양이 요구됩니다.

	Android 스마트폰	iOS 스마트폰
최소 OS 요구사항	Android 10.0 이상	iOS 15.0 이상

3.4 임상시험 방법 및 절차

본 임상시험에 참여하는 연구대상자는 시험군, 대조군으로 1:1 무작위 배정됩니다. 시험군은 MORA Cure 디지털치료기기를 사용하는 군이며, 대조군은 운동교육 및 자가운동 자료를 제공하는 군입니다.
첫 번째 방문(V1)에는 동의 취득 및 선정/제외 기준 확인을 진행합니다. 두 번째 방문(V2, 0wk)에 무작위 배정을 진행하고 시험군은 임상시험용 의료기기 처방 및 설치, 대조군은 운동교육 및 자가운동 자료를 제공합니다. 세 번째 방문(V3, 4주), 네 번째 방문(V4, 8주), 다섯 번째 방문(V5, 12주) 에는 각종 검사 및 평가가 이루어집니다.
시험군(임상시험용 의료기기): 임상시험용 의료기기에 접속하면 “오늘 할일”에 재활운동 프로그램과 수행해야하는 시간 및 인지행동치료 등 제공됩니다. 또한 수행률을 확인할 수 있습니다.

대조군(운동교육 및 자가운동자료): 별도로 대상자에게 제공되며, 1~4주차, 5~8주차 프로그램으로 구성되어 있습니다. 운동동작 예시와 설명을 담고 있습니다.

시험 진행 시 귀하는 병력, 수술력, 선행 약물, 병용 약물, 통증 강도, 이상사례 및 부작용 등을 질문받고 대답하게 됩니다.

귀하께서 받게 되는 검사와 평가로는 신체검진(슬개골 압박 검사, 슬개골 주변 압통 검사, 관절 종창 징후, 무릎관절 굴곡구축 및 굴곡제한의 정도)가 있으며 무릎 X-ray, 각종 설문지(Kujala, EQ-5D-5L, PHQ-9, PCS, 전반적인 체감 회복 정도)가 있습니다.

Kujala는 최근 무릎상태를 확인하는 평가도구이며 13개의 문항으로 구성되어 있습니다. EQ-5D-5L은 방문일에 건강상태를 물어보게되며 운동능력, 자기관리, 일상활동, 통증/불편, 불안/우울 관련 확인하는 평가도구로 5개의 문항으로 구성되어 있습니다. PHQ-9는 우울한 정도를 알아보기 위한 평가도구로 지난 2주간 느꼈던 9개의 항목에 답하는 평가도구입니다. PCS는 통증을 느낄 때의 사고 감정에 대해 물어보는 평가도구이며 13개의 항목으로 구성되어 있습니다. 전반적인 체감 회복 정도는 통증 강도를 물어보게 되며, 각 설문지 작성은 5분이내로 소요될 예정입니다.

PHQ-9 설문지 결과 20점 이상이라면 정신건강의학과에 협진이 이루어질 수 있습니다.

3.5 임상시험 참여 기간 및 대상자 수

본 임상시험의 참여 기간은 12주~16주입니다. 귀하는 병원에 총 4~5번 방문하게 됩니다.

총 목표 연구대상자 수는 216명입니다.

4 임상시험 참여에 따른 위험성과 이익

4.1 임상시험 참여 시 발생할 수 있는 불편 또는 위험성

임상시험용 의료기기 (MORA Cure)의 사용 중 다음의 부작용이 일시적으로 발생할 수 있습니다.

- 일시적인 무릎 통증의 악화
- 운동 중/운동 후 일시적인 근육통, 경련(cramp), 어지러움 등
- 무릎 통증의 패턴 (강도, 부위)의 변화
- 일시적인 기분 및 사고 패턴의 변화
- 그 밖의 예상하지 못한 부작용

연구자는 귀하에게 발생 가능한 부작용에 대해 자세히 안내하고, 연구대상자가 궁금한 것이 있다면 언제든지 자세히 설명할 것입니다. 귀하는 예정된 정기 방문일이 아니더라도 연구자의 판단 하에 병원을 방문하여 진료받을 수 있습니다.

4.2 임상시험 참여 시 받을 수 있는 이익

귀하는 임상시험용 의료기기 혹은 운동교육 및 자가운동을 통해 무릎 통증의 개선을 기대할 수 있습니다. 단, 효과가 없을 수도 있습니다. 귀하가 본 연구에 참여함으로서 귀하에게 주어지는 이익이 없을 수도 있지만, 미래에 귀하와 같은 질병 또는 상태에 있는 환장에게 혜택이 주어질 수 있습니다.

4.3 대체 치료방법

무릎 통증에 대해서 본 임상시험 의료기기, 교육, 자가운동, 인지행동치료 이외에 온열 치료, 견인 치료, 도수 치료, 약물 치료 등이 통증 완화의 효과가 있을 수도 있다고 알려져 있습니다.

4.4 대상자가 준수하여야 할 사항

귀하께서 임상시험에 참여하기로 결정하였다면 다음을 준수하여야 합니다.

1. 미리 정해진 방문일을 준수합니다.
2. 배정된 치료를 성실하게 수행합니다.
3. 연구자의 질문 및 설문지 등에 사실대로 답변합니다.
4. 매 방문마다 복용하는 모든 약(처방전 없이 구입할 수 있는 약 포함)을 담당 연구자에게 알립니다.
5. 건강에 변화가 있거나 이상 사례나 부작용의 발생, 그 밖의 우려가 있다면 연구자에게 알려야 합니다.

4.5 대상자에게 연구 참여 기간 동안 금지되는 사항

임상시험 기간(V1~V5) 동안 다음의 약물, 치료, 시술, 수술 등을 금지하여야 합니다.

1. 관절강내 스테로이드 등 무릎 관절 내 혹은 주변의 주사 치료
2. 관절경 수술, 교정 절골술, 인공관절 수술 등 시험자가 승인하지 않은 시술 및 수술
3. 통증 조절 목적의 마약성 진통제 복용
4. 무릎에 적용하는 외용제 혹은 패치
5. 시험자가 승인하지 않은 기타 관절 통증 완화 또는 조절 목적의 약물 및 치료
6. 시험자 판단 하에 관절에 무리가 가는 육체적인 운동

4.6 임상시험 관련 새로운 정보를 알게 된 경우

연구자 및 의뢰자는 임상시험 계속 참여와 관련된 중요한 새로운 정보를 제때에 귀하에게 제공할 것입니다. 이러한 정보에는 예기치 않은 이상반응, 빈번하게 발생하는 부작용 등이 포함될 수 있습니다. 귀하는 이러한 정보를 제공받은 뒤 언제든지 자의로 임상시험에의 참여를 중단할 수 있습니다.

5 임상시험 관련 비용 부담

5.1 대상자가 부담하여야 하는 비용

귀하가 임상시험 참여를 위해 따로 부담하여야 하는 비용은 없습니다. 단, 임상시험 기간 동안 연구와 관련 없는 진료나 입원, 수술을 받은 경우는 귀하가 스스로 금액을 부담하여야 합니다. 임상시험이 모두 종료된 이후의 진료, 입원, 수술의 경우에도 스스로 금액을 부담하여야 합니다.

5.2 대상자가 받게 될 금전적 보상

귀하는 매 방문마다 50,000원의 교통비를 지급받게 됩니다. 교통비는 매 방문마다 지급되며, 첫 번째 방문과 두 번째 방문이 함께 이루어지는 경우에는 100,000원이 지급됩니다. 임상시험에서 중도 탈락되는 경우에도 마지막 방문까지는 교통비를 지급받을 수 있습니다. 귀하가 지급받게 될 교통비는 귀하가 임상시험에 참여함으로써 얻는 이익이나 혜택으로 간주되지 않습니다.

교통비 외에 본 임상시험 참여로 인한 별도의 사례나 보상은 없습니다. 단, 임상시험 중 이루어지는 진료, 임상시험용 의료기기의 사용, 운동치료 및 교육, 각종 검사(X-ray 검사, 신체 검진, 설문지 검사 등)에 대한 비용은 의뢰자인 에버엑스 주식회사가 부담합니다.

6 임상시험 관련 피해보상

임상시험 참여 중에 귀하에게 발생하는 피해의 보상은 배부된 **피해자 보상 규약** 및 의뢰자가 임상시험 시작 전 가입한 **임상시험배상보험**에 근거하여 이루어질 것입니다.

귀하는 임상시험 진행 중 피해 혹은 손상이 발생한 경우 9 문의처~~문의처~~에 연락하여 상담하고 보상 신청을 할 수 있습니다. 아울러, 연구자는 귀하에게 피해 혹은 손상이 발생한 경우 이를 귀하에게 알리고 보상 관련 절차 등을 안내할 것입니다.

서식 지정
글) Noto

7 임상시험 참여 중단

7.1 자발적인 참여 중단

귀하는 자유로운 의사로 본 임상시험에의 참여를 중단할 수 있으며, 임상시험 참여에 동의하고 서명한 이후라도 언제든지 참여 의사를 철회할 수 있습니다. 연구 참여를 중단하는 것은 어떠한 불이익도 초래하지 않을 것이며, 향후 치료 과정에 어떠한 영향도 미치지 않을 것입니다.

7.2 임상시험 중단 및 그 대책

귀하는 다음의 경우 임상시험에서 중도 탈락됩니다.

1. 임상시험 기간 동안 시험자의 지시 없이 임상시험 결과에 영향을 미칠 수 있는 약물 투여, 시술, 수술, 치료를 받은 경우
2. 선정/제외기준에 적합하지 않은 연구대상자가 임상시험에 참여한 경우
3. 연구대상자의 추적관찰이 불가능한 경우(follow-up loss)
4. 이상사례로 인하여 시험자가 해당 연구대상자의 임상시험 참여를 중단해야 한다고 판단한 경우
5. 연구대상자 또는 연구대상자의 대리인이 임상시험 중단을 요구하는 경우(동의 철회)
6. 기타 사유로 시험자가 해당 연구대상자의 임상시험 진행이 적합하지 않다고 판단하는 경우

중도 탈락되는 경우, 혹은 임상시험이 중단되거나 조기종료되는 경우 귀하는 임상시험 참여를 중단하게 됩니다. 이 경우 연구자는 연구대상자에게 신속하게 그 취지를 통보하고 적절한 치료 및 사후 처리를 진행할 것입니다.

8 개인정보의 보호

임상시험에 참여하기 앞서 연구자는 귀하에게 개인정보 수집·이용에 대한 동의를 받게 됩니다. 단, 임상시험용 의료기기를 사용하는 사람에 한하여 추가적으로 소프트웨어 의료기기 (애플리케이션) 설치 및 회원가입 시 개인정보 수집·이용, 제3자 제공에 대한 동의 여부를 추가적으로 확인합니다.

본 임상시험을 위하여 귀하의 개인정보 및 민감정보가 수집되지만, 모든 데이터는 엄격하게 비밀로 유지되며 보호받게 됩니다. 귀하의 개인 정보 및 민감정보는 비밀로 유지되며, 검사 결과 등에는 이름과 개인정보 대신 익명 처리한 후 독립적으로 부여한 연구용 번호를 부여하여 사용할 것입니다. 연구자는 이 목록을 안전하게 보관할 것이며, 본 임상시험의 결과가 출판되더라도 귀하의 개인정보가 노출되는 일은 없습니다.

임상시험 진행 중 및 임상시험 종료 후에도 모니터요원, 점검자, 연구윤리위원회 및 식품의약품안전처장, 보건복지부장관 등이 관계 법령에 따라 연구의 절차와 자료의 품질을 검증하기 위하여 귀하의 신상에 관한 비밀이 보호되는 범위에서 연구 기록을 열람할 수 있습니다. 이러한 연구 기록의 열람은 귀하 또는 대리인이 서명 동의가 있는 경우에만 허용됩니다.

본 임상시험이 진행되면서 얻어진 귀하의 모든 신상에 대한 기록은 다른 사람에게 알려지지 않도록 비밀로 보장될 것입니다. 연구대상자의 정보나 임상시험의 결과가 공개 사이트에 등록되거나 출판되는 등 제3자에게 제공될 수 있으나 귀하의 신상과 신상을 파악할 수 있는 기록은 비밀로 보호됩니다.

개인정보보호법 제4조(정보주체의 권리)에 따라 귀하는 개인정보의 처리에 관한 정보를 제공받을 권리, 개인정보의 처리에 관한 동의 여부, 동의 범위 등을 선택하고 결정할 권리, 개인정보의 처리 여부를 확인하고

개인정보에 대하여 열람을 요구할 권리, 개인정보의 처리 정지, 정정 삭제 및 파기를 요구할 권리, 개인정보의 처리로 인하여 발생한 피해를 신속하고 공정한 절차에 따라 구제받을 권리가 있습니다.

임상시험실시기관 측에서 수집 및 이용하는 개인정보 내역, 제3자 측에 제공되는 개인정보 내역에 대해서는 본 피험자 설명서 및 동의서 중 "동의서" 부분에 자세히 기술되어 있습니다. 귀하는 개인정보 수집·이용 및 제 3자 제공에 대한 동의를 거부할 수 있는 권리가 있으며, 동의를 거부한 경우에는 본 임상시험에 참여하실 수 없습니다.

9 문의처

임상시험과 연구대상자의 권익에 대하여 추가적인 정보를 얻고자 하거나 임상시험과 관련이 있는 손상이 발생한 경우 다음에 연락할 수 있습니다.

**임상시험에 대하여 상의할 연구자의 연락처**	**연구대상자의 권익에 대해 상의할 생명윤리심의위원회 또는 임상연구윤리센터 연락처**
시험 책임자(공란): 공란<br>24시간 연락처: 공란	공란

10 동의 절차

피험자의 동의를 얻기 전에 시험책임자 또는 시험책임자의 위임을 받은 의사, 치과의사, 한의사는 피험자 또는 피험자의 대리인이 임상시험의 세부 사항에 대해 질문하고 해당 임상시험의 참여 여부를 결정할 수 있도록 충분한 시간과 기회를 주어야 하며, 임상시험과 관련한 모든 질문에 대하여 피험자 또는 피험자의 대리인에게 성실하게 답변하여야 합니다.

연구대상자의 임상시험 참여 전에 연구대상자 또는 연구대상자의 대리인과 동의를 받은 시험책임자 또는 시험책임자의 위임을 받은 의사, 치과의사, 한의사는 동의서에 서명하고, 해당 날짜를 자필로 적어야 합니다.

연구대상자의 이해 능력·의사표현능력의 결여 등으로 친권자 또는 후견인 등의 동의를 받아야 참여가 가능한 경우, 연구자는 연구대상자에게 연구대상자 자신이 이해할 수 있는 정도까지 임상시험에 관한 정보를 줄 것이며, 가능하면 연구대상자는 동의서에 자필로 서명하고 날짜를 적도록 하여야 합니다.

연구대상자 또는 연구대상자의 대리인이 동의서 서식, 연구대상자 설명문, 그 밖의 문서화된 정보를 읽을 수 없는 경우에는 참관인이 동의를 받는 모든 과정에 참석하여야 합니다. 이 경우 시험책임자 또는 시험책임자의

위임을 받은 자는 동의서 서식, 연구대상자설명서 및 그 밖의 문서화된 정보를 연구대상자 또는 연구대상자의 대리인에게 읽어 주고 설명하여야 하며, 연구대상자 또는 연구대상자의 대리인은 임상시험 참여를 구두로 동의하고 가능하다면 동의서에 자필로 서명하고 해당 날짜를 적고, 참관자가 동의서에 자필로 서명하고 해당 날짜를 적어야 하며, 참관자는 동의서에 서명하기 전에 동의서와 연구대상자설명서 및 그 밖의 문서화된 정보가 정확하게 연구대상자나 연구대상자의 대리인에게 설명되었는지 여부, 이들이 해당 사실을 이해하였는지 여부 및 동의를 얻는 과정이 연구대상자나 연구대상자의 대리인의 자유의사에 따라 진행되었는지 여부를 확인하여야 합니다.

연구자는 임상시험에 참여하기 전에 동의서의 사본 및 연구대상자에게 제공된 그 밖의 문서화된 정보의 사본을 연구대상자 또는 연구대상자의 대리인에게 주어야 하며, 임상시험 도중에 동의서 서식이 변경된 경우 연구자는 연구대상자나 연구대상자의 대리인에게 변경동의서의 사본을 주어야 하고, 이미 연구대상자에게 제공된 문서 정보의 변경이 있는 경우에는 해당 변경 문서의 사본을 주어야 합니다.

연구대상자 동의서

다음 각 항목에 대해 동의하면 “예”, 동의하지 않으면 “아니오”에 표기하십시오.

<table>
<tr><th>번호</th><th>내용</th><th>예</th><th>아니오</th></tr>
<tr><td>1</td><td>본인은 본 임상시험에 대한 모든 정보들에 관하여 담당의사로부터 자세하게 설명을 듣고 충분히 이해하였습니다.</td><td>☐</td><td>☐</td></tr>
<tr><td>2</td><td>본인은 또한 대상자동의 설명문을 읽어 보았으며, 그 내용을 충분히 이해하였습니다.</td><td>☐</td><td>☐</td></tr>
<tr><td>3</td><td>본인은 임상시험 기간 중 언제라도 개인적인 사유 등으로 지속적인 참여를 중도에 거부하거나 자유로이 참가를 중단할 수 있으며, 이로 인해 진료 및 기타 어떠한 불이익도 받지 않음을 알고 있습니다.</td><td>☐</td><td>☐</td></tr>
<tr><td>4</td><td>본인은 임상시험 지속 참여 의지에 영향을 줄 수 있는 새로운 정보가 수집되면 적시에 알려질 것이라는 사실을 확인하였습니다.</td><td>☐</td><td>☐</td></tr>
<tr><td>5</td><td>본인은 임상시험 관련하여 의문이 있을 경우에는 언제라도 시험담당자 또는 해당기관의 임상시험심사위원회에 문의할 수 있으며 연락처는 본문의 [21. 담당자 연락처]와 같음을 확인하였습니다.</td><td>☐</td><td>☐</td></tr>
<tr><td>6</td><td>본인은 모니터요원, 점검을 실시하는 자, 심사위원회 및 식품의약품안전처장이 관계 법령에 따라 임상시험의 실시 절차와 자료의 품질을 검증하기 위하여 피험자의 신상에 관한 비밀에 보호되는 범위에서 피험자의 의무기록을 열람하는 데에 동의합니다.
※ 해당 항목에 “예”에 체크하여 동의한 경우에만 열람이 가능합니다.</td><td>☐</td><td>☐</td></tr>
<tr><td>7</td><td>본인은 임상시험 참여 여부에 따라 공개 사이트에 임상시험 결과 정보가 등록될 수 있음과 출판될 수 있다는 것에 동의합니다.
※ 피험자의 신상을 파악할 수 있는 모든 기록은 비밀로 보호됩니다.
※ 임상시험의 결과가 출판될 경우 피험자의 신상은 비밀로 보호됩니다.</td><td>☐</td><td>☐</td></tr>
<tr><td>8</td><td>본인은 임상시험실시기관 측에 하기 개인정보를 제공하는 것에 관한 내용을 확인하였으며 정보 수집 및 이용에 대하여 동의합니다.
※ 귀하는 정보 수집 및 이용에 대한 동의를 거부할 수 있으나, 동의를 거부할 경우 본 임상시험에 참여할 수 없습니다.
▶ 개인정보 수집·이용 내역<table><tr><td>개인정보항목</td><td>생년월일, 성별</td></tr><tr><td>수집·이용목적</td><td>타 임상시험 중복참여 확인, 임상시험 진행 및 모니터링, 결과보고, 사후 관리, 점검, 실태조사, 출판</td></tr><tr><td>보유·이용기간</td><td>식품의약품안전처의 품목허가일 또는 시험의 완료일로부터 3 년</td></tr></table></td><td>☐</td><td>☐</td></tr>
<tr><td>9</td><td>본인은 임상시험실시기관 측에 하기 민감정보를 제공하는 것에 관한 내용을 확인하였으며 정보 수집 및 이용에 대하여 동의합니다.
※ 귀하는 정보 수집 및 이용에 대한 동의를 거부할 수 있으나, 동의를 거부할 경우 본 임상시험에 참여할 수 없습니다.
▶ 민감정보 수집·이용 내역<table><tr><td>민감정보항목</td><td>병력, 약물력, 치료력, 신장, 체중 및 신체검진 자료, 병용약물 및 병용치료에 대한 자료, 이상사례, 무릎 X-ray, 통증 강도 평가자료, 설문지 평가자료</td></tr><tr><td>수집·이용목적</td><td>타 임상시험 중복참여 확인, 임상시험 진행 및 모니터링, 결과보고, 사후 관리, 점검, 실태조사, 출판</td></tr><tr><td>보유·이용기간</td><td>식품의약품안전처의 품목허가일 또는 시험의 완료일로부터 3 년</td></tr></table></td><td>☐</td><td>☐</td></tr>
</table>

<table>
<tr><td>10</td><td>본인은 제 3 자 측에 하기 개인정보를 제공하는 것에 관한 내용을 확인하였으며 이에 대하여 동의합니다.
※ 귀하는 정보 수집 및 이용에 대한 동의를 거부할 수 있으나, 동의를 거부할 경우 본 임상시험에 참여할 수 없습니다.
▶ 개인정보 제 3 자 제공 내역<table><tr><td>제공받는 자</td><td>식품의약품안전처, 시험의뢰자, 임상시험심사위원회, 타 임상시험기관, 모니터 요원, 임상시험 중복참여 여부를 확인하기 위해 식품의약품안전처장이 정하는 홈페이지 운영 기관</td></tr><tr><td>제공 목적</td><td>타 임상시험 중복참여 확인, 임상시험 진행 및 모니터링, 결과보고, 사후 관리, 점검, 실태조사, 출판</td></tr><tr><td>제공 항목</td><td>생년월일, 성별</td></tr><tr><td>보유·이용 기간</td><td>식품의약품안전처의 품목허가일 또는 시험의 완료일로부터 3 년</td></tr></table></td><td>☐</td><td>☐</td></tr>
<tr><td>11</td><td>본인은 제 3 자 측에 하기 민감정보를 제공하는 것에 대한 내용을 확인하였으며, 이에 대하여 동의합니다.
※ 귀하는 정보 수집 및 이용에 대한 동의를 거부할 수 있으나, 동의를 거부할 경우 본 임상시험에 참여할 수 없습니다.
▶ 민감정보 제 3 자 제공 내역<table><tr><td>제공받는 자</td><td>식품의약품안전처, 시험의뢰자, 임상시험심사위원회, 타 임상시험기관, 모니터 요원, 임상시험 중복참여 여부를 확인하기 위해 식품의약품안전처장이 정하는 홈페이지 운영 기관</td></tr><tr><td>제공 목적</td><td>타 임상시험 중복참여 확인, 임상시험 진행 및 모니터링, 결과보고, 사후 관리, 점검, 실태조사, 출판</td></tr><tr><td>제공 항목</td><td>병력, 약물력, 치료력, 신장, 체중 및 신체검진 자료, 병용약물 및 병용치료에 대한 자료, 이상사례, 무릎 X-ray, 통증 강도 평가자료, 설문지 평가자료</td></tr><tr><td>보유·이용 기간</td><td>식품의약품안전처의 품목허가일 또는 시험의 완료일로부터 3 년</td></tr></table></td><td>☐</td><td>☐</td></tr>
<tr><td>12</td><td>이에 본인의 자유로운 의사에 따라 본 임상시험에 참여할 것을 동의하며, 본 동의서의 사본 1 부를 수령합니다.</td><td>☐</td><td>☐</td></tr>
</table>

<table>
<tr><th>구 분</th><th>성 명</th><th>서 명</th><th>서 명 날 짜</th></tr>
<tr><td>연구대상자</td><td></td><td></td><td>년 월 일</td></tr>
<tr><td rowspan="2">대상자의 대리인
(해당되는 경우)</td><td></td><td></td><td>년 월 일</td></tr>
<tr><td colspan="3">대상자와의 관계: ____________________
구체적인 사유: ____________________</td></tr>
<tr><td>시험책임자
(또는 위임된 시험자)</td><td></td><td></td><td>년 월 일</td></tr>
</table>
